# Retinoic acid modulates prolactin receptor expression and prolactin-induced STAT-5 activation in breast cancer cells in vitro

**DOI:** 10.1038/sj.bjc.6690034

**Published:** 1999-01

**Authors:** M Widschwendter, A Widschwendter, T Welte, G Daxenbichler, A G Zeimet, A Bergant, J Berger, J-P Peyrat, S Michel, W Doppler, C Marth

**Affiliations:** Department of Gynecology and Obstetrics, University of Innsbruck, Anichstrasse 35, Innsbruck, A-6020, Austria; Department of Medical Biochemistry, University of Innsbruck, Anichstrasse 35, Innsbruck, A-6020, Austria; Centre Oscar Labret, Lille, F-59020, France; CIRD Galderma, Sophia Antipolis, Valbonne Cedex, F-06565, France

**Keywords:** breast cancer, prolactin, prolactin receptor, retinoids, retinoic acid, STAT-5

## Abstract

Two recent papers demonstrate that prolactin plays an important role in the induction and progression of mammary tumours. Retinoids have been shown to be potent inhibitors of breast carcinogenesis. We studied expression of prolactin receptor mRNA in human breast cancer cell lines MCF-7, SKBR-3, T47D and BT-20 treated with and without retinoids using Northern blot and a quantitative polymerase chain reaction (PCR) method. In all cell lines, all-*trans*- and 9-*cis*-retinoic acid, as well as the retinoic acid receptor γ (RAR-γ) selective agonists CD2325 and CD437 (1 μM), were able to down-regulate prolactin receptor. After 1 h, a significant reduction was detectable and maximal effect was achieved after 24 h of treatment. Pretreatment with retinoic acid also reduced the prolactin-/prolactin receptor-dependent signal transduction and activation of transcription 5 (STAT-5) activation in T47D cells. Cycloheximide failed to abrogate the retinoic acid-induced decline in prolactin receptor mRNA levels, indicating that this effect was not dependent upon continuing protein synthesis. Similarly, no change in the stability of prolactin receptor mRNA was observed during 12 h of retinoic acid treatment. In conclusion, our results demonstrate that retinoids are able to inhibit the expression of prolactin receptor message, which encodes an important growth factor receptor in breast cancer cells. This action could be responsible for the anti-tumour effects of retinoids. © 1999 Cancer Research Campaign


*British Journal of Cancer* (1999) **79**(2), 204–210
© 1999 Cancer Research Campaign

### Retinoic acid modulates prolactin receptor expression and prolactin-induced STAT-5 activation in breast cancer cells in vitro

**M Widschwendter[1], A Widschwendter[1], T Welte[2], G Daxenbichler[1], AG Zeimet[1], A Bergant[1], J Berger[1], J-P Peyrat[3], S Michel[4], W Doppler[2] and C Marth[1]**

Departments of [1]Gynecology and Obstetrics and [2]Medical Biochemistry, University of Innsbruck, Anichstrasse 35, A-6020 Innsbruck, Austria; and [3]Centre Oscar Labret, F-59020 Lille, France; and [4]CIRD Galderma, Sophia Antipolis, F-06565 Valbonne Cedex, France

**Summary** Two recent papers demonstrate that prolactin plays an important role in the induction and progression of mammary tumours. Retinoids have been shown to be potent inhibitors of breast carcinogenesis. We studied expression of prolactin receptor mRNA in human breast cancer cell lines MCF-7, SKBR-3, T47D and BT-20 treated with and without retinoids using Northern blot and a quantitative polymerase chain reaction (PCR) method. In all cell lines, all-*trans*- and 9-*cis*-retinoic acid, as well as the retinoic acid receptor γ (RAR-γ) selective agonists CD2325 and CD437 (1 μM), were able to down-regulate prolactin receptor. After 1 h, a significant reduction was detectable and maximal effect was achieved after 24 h of treatment. Pretreatment with retinoic acid also reduced the prolactin-/prolactin receptor-dependent signal transduction and activation of transcription 5 (STAT-5) activation in T47D cells. Cycloheximide failed to abrogate the retinoic acid-induced decline in prolactin receptor mRNA levels, indicating that this effect was not dependent upon continuing protein synthesis. Similarly, no change in the stability of prolactin receptor mRNA was observed during 12 h of retinoic acid treatment. In conclusion, our results demonstrate that retinoids are able to inhibit the expression of prolactin receptor message, which encodes an important growth factor receptor in breast cancer cells. This action could be responsible for the anti-tumour effects of retinoids.

**Keywords:** breast cancer; prolactin; prolactin receptor; retinoids; retinoic acid; STAT-5

Endocrine therapy is a hallmark of breast cancer treatment. The principle of such therapies is primarily based on antagonism of oestradiol, long considered the only mitogen for human breast cancers. In addition to steroids, prolactin (PRL) plays an important role in the induction and progression of mammary tumours (Welsch and Nagasawa, 1997; Vonderhaar, 1989; Bhatavdekar and Patel, 1997).

Wennbo and co-workers (Wennbo et al, 1997) showed – using transgenic mice overexpressing the bovine growth hormone (GH) and mice overexpressing the rat PRL – that the prolactin receptor (PRL-R) alone is sufficient for induction of mammary carcinomas in mice, whereas activation of the GH receptor is not sufficient for mammary tumour formation.

In vitro, primary cultures of human mammary epithelial cells display an absolute requirement for prolactin for growth and passage on tissue culture plastic or inside collagen gels (Malarkey et al, 1983). Prolactin induces the phosphorylation of tyrosine 694 of STAT-5 (*s*ignal *t*ransduction and *a*ctivation of *t*ranscription), presumably as a consequence of activation of the JAK2 tyrosine kinase (Rui et al, 1994), and this has been demonstrated to be a prerequisite for DNA binding and gene activation (Gouilleux et al, 1994; Goffin and Kelly, 1996 and references therein).

*Received 16 January 1998*
*Revised 8 June 1998*
*Accepted 16 June 1998*

*Correspondence to*: M Widschwendter, Department of Obstetrics and Gynecology, University of Innsbruck, Anichstrasse 35, A-6020 Innsbruck, Tirol, Austria

Recently, it was shown that breast cancer cell lines produce and secrete PRL and that an auto- or paracrine loop mediated by PRL and PRL-R may be involved in the regulation of proliferation (Ginsburg and Vonderhaar, 1995). Not only in benign but also in malign breast tissue, mRNA for PRL-R and PRL could be found (Clevenger et al, 1995; Shaw-Bruha et al, 1997). These recent data may be the explanation why dopaminagonists – inhibitors of the pituitary prolactin secretion – only showed modest effects in breast cancer (Anderson et al, 1993). In contrast, it could be shown that retinoic acid (RA) treatment, not only in vivo but also in vitro, was able to interfere with the tumour-stimulating activity of PRL: rats given *N*-methyl-*N*-nitrosourea and subsequently treated with the prolactin secretion-stimulating drug haloperidol responded with a significant increase in mammary carcinoma development when compared with control rats. RA treatment of haloperidol-treated rats significantly ($P$<0.001) blocked the PRL-mediated stimulatory effect on mammary carcinoma development (Welsch et al, 1984). In this model, retinoid treatment has no effect on PRL serum levels, indicating that the anti-tumour effect of retinoids must occur at the level of tumour cells stimulated by PRL.

In vitro, retinoids have been shown to inhibit the growth of human breast cancer cells (Koga and Sutherland, 1991; Marth et al, 1993; Gottardis et al, 1996; Widschwendter et al, 1997). Retinoids have been shown to protect against chemically induced breast carcinoma in animals and to reduce the proliferation of cultured breast cancer cells (Marth et al, 1986).

Retinoids are known to possess antiproliferative, differentiative and immunomodulatory properties. The key molecules in retinoid action are the binding proteins CRABP I and II (cellular retinoic acid-binding protein), the retinoid receptors (RAR-α, RAR-β,

RAR-γ) and retinoid X receptors (RXR-α, RXR-β, RXR-γ), which are part of the steroid/thyroid hormone receptor superfamily (Sporn et al, 1994). A growing body of evidence from clinical research supports the concept that retinoids are useful substances in the prevention and treatment of cancer. The RA-provoked growth effects were synergistically amplified by a combination with interferon γ (IFN-γ), and this was accompanied by up-regulation of the mRNA for nuclear receptor RAR-γ (retinoic acid receptor-γ) (Widschwendter et al, 1995, 1996). Similar effects were also achieved with the RAR-γ selective agonists CD2325 and CD437 (Widschwendter et al, 1997). In animals, administration of retinoids inhibits the initiation and promotion of mammary tumours induced by carcinogens (Moon and Mehta, 1990; Costa, 1993).

Anzano et al (1994) showed 9-*cis*-RA alone or in combination with tamoxifen is a very potent inhibitor of mammary carcinogenesis induced by *N*-nitroso-*N*-methylurea in Sprague–Dawley rats. On the basis of these results, several clinical trials with retinoids have been carried out: tamoxifen and retinyl acetate caused an objective response rate in 39% of 33 patients with advanced breast cancer. In a phase I/II trial, treatment with tamoxifen plus fenretinide resulted in improvement or disease stabilization in 12 out of 15 patients (80%), with no significant adverse effects (Cobleigh et al, 1993). Fenretinide was well tolerated in a preventive trial for contralateral breast cancer comprising 2972 patients with minor side-effects observed during 5 years of treatment (Formelli et al, 1993; Costa et al, 1994). Two large adjuvant studies comparing tamoxifen plus fenretinide with tamoxifen alone started last year.

The mechanism of anti-tumour effects of retinoids is, however, not fully understood, and we were interested in whether retinoids may interact with PRL-mediated effects. We, therefore, studied the modulation of expression of PRL-R mRNA by retinoids in the human breast cancer cell lines MCF-7, SKBR-3, T47D and BT-20 applying Northern blot and a quantitative polymerase chain reaction (PCR) method. Because one of the most important mediators of PRL-regulated genes is STAT-5 (Welte et al, 1994), we were interested in whether this transcription factor is also modulated by RA.

### MATERIALS AND METHODS

### Reagents

9-cis-Retinoic acid and ATRA (all-*trans*-retinoic acid) were kindly provided by Professor Bollag (Hoffmann-La Roche, Basle, Switzerland). The RAR-γ selective agonists CD2325 and CD437 were donated by Professor Reichert (CIRD Galderma, Sophia Antipolis, France). For all experiments, 1 mM solutions were prepared in DMSO and further diluted in complete culture medium.

### Cell culture

The MCF-7, BT-20, SKBR-3 and T47D human breast cancer cell lines were cultured as described previously (Widschwendter et al, 1995). The cell lines used in this study were generous gifts from Dr GC Buehring, School of Public Health, Berkley, CA, USA, and Dr NE Hynes, F Miescher Institute, Basle, Switzerland. Briefly, the cells were maintained in modified Eagle medium (MEM) containing 10% fetal bovine serum (both from Eurobio, Paris, France), 100 U $ml^{-1}$ penicillin and 100 μg $ml^{-1}$ streptomycin. Cells were maintained at 37°C in a humidified atmosphere of 5% carbon dioxide in air. Exponentially growing cells ($1\times10^6$) were plated in 25 ml medium per 100-$cm^2$ flask. After reaching 80% confluence, cells were treated with substances or vehicle alone as control for indicated time and concentrations, detached with the help of trypsin (0.05%)–EDTA (0.02%) in Dulbecco’s phosphate-buffered saline (PBS), washed and pelleted.

### Northern blot analysis

Northern blot analysis was carried out as recently described (Widschwendter et al, 1995). Briefly, total cellular RNA was extracted by the guanidine thiocyanate method. Ten micrograms of total RNA mixed with ethidium bromide was run on denaturing 1% agarose–formaldehyde gels and transferred to nylon membranes (Stratagen Flash Nylon Membranes, La Jolla, CA, USA) by Northern blotting. The sheet thus prepared was fixed and photographed under UV light (to demonstrate comparable RNA levels) and hybridized with the digoxigenin-labelled 310-bp DNA fragment which codes for the extracellular domain of the PRL-R (described below). Detection of digoxigenin-labelled nucleic acids by chemiluminescence enzyme immunoassay on nylon membranes was carried out following manufacturer’s instructions (DIG Luminescent Detection Kit, Boehringer Mannheim Biochemica, Vienna, Austria). Filters were exposed to autoradiographic films (Hyperfilm, Amersham, CEAB, Sweden) for 5 h.

### Quantitation of RNA

Total RNA was isolated as previously described (Widschwendter et al, 1995). Quantitation of RNA was performed as described previously (Doppler et al, 1991). Briefly, an aliquot corresponding to 400 ng of RNA was reverse transcribed. PCRs were performed in a volume of 25 μl. The primers used were: 5′-TGC ACC ACC AAC TGC TTA GCA-3′ and 5′-GAA GTC AGA GGA GAC CAC CTG-3′ for glyceraldehyde phosphate dehydrogenase (GAPDH), yielding a 405-bp fragment spanning positions 513–918 of human cDNA (Tso et al, 1985), and 5′-ACT TAC ATA GTT CAG CCA GAC C-3′ and 5′-TGA ATG AAG GTC GCT GGA CTC C-3′ for PRL-R, yielding a 310-bp fragment spanning positions 363–673 of human cDNA and recognizing the extracellular form of PRL-R (Boutin et al, 1989). Thirty cycles were performed in a thermocycler. The amplification profile involved 30 s at 94°C, 15 s at 95°C, 75 s at 55°C and 1 min at 73°C. From the 21st cycle, the 73°C step was extended by 10 s every cycle. PCR products were run on a 2% agarose gel containing ethidium bromide. Quantification of yield was performed by video imaging using the Bioprofil Program (Version 4.01; Vilber Lourmat).

Results of PRL-R were then normalized against the amount of GAPDH cDNA detected in the corresponding samples.

### Statistics

Differences in the median yield of cDNA were analysed by the Wilcoxon *U*-test (Sachs, 1992).

### PRL-R protein determination

PRL-R protein was determined by incubating membrane proteins of MCF-7 cells with 100 000 c.p.m. iodinated human growth hormone, in the presence or absence of a 1000-fold excess of

© Cancer Research Campaign 1999

unlabelled ovine PRL according to an assay described by Bonneterre et al 1987). Specific binding was calculated as the difference between the c.p.m. bound in the absence and the presence of the excess unlabelled PRL.

### Whole-cell extracts

Cells were scraped off the dishes in cold PBS, pelleted and extracted by three cycles of freezing and thawing in 2–3 volumes of 10 mM sodium phosphate, pH 7.4, 1 mM EDTA, 1 mM dithiothreitol, 400 mM potassium chloride, 10% glycerol supplemented with 5 μg $ml^{-1}$ aprotinin, 5 μg $ml^{-1}$ leupeptin, 1 μM pepstatin, 1 mM phenylmethylsulphonyl fluoride (PMSF), 5 μM sodium fluoride, and 0.5 μg $ml^{-1}$ okadeic acid. Extracts were clarified at 17 000 ***g*** for 15 min. Protein concentrations were 2–7 μg $\mu l^{-1}$.

### Electrophoretic mobility shift assays

Assays were performed by a method similar to that described by Welte et al (1994). Oligonucleotides were purified by polyacrylamide gel electrophoresis, radioactively labelled with [$\gamma$-$^{32}$P]ATP (>6000 Ci $mmol^{-1}$) and T4 polynucleotide kinase, and purified by phenol extraction and Sephadex G50 chromatography. After treatment for 5 min at 95°C, complementary oligonucleotides were annealed. Cellular extracts were incubated with oligonucleotide (25 000 c.p.m., 10–35 fmol) on ice (30 min) in a 20-μl reaction volume containing 10 mM Hepes, pH 7.6, 2 mM sodium phosphate, 0.25 mM EDTA, 1 mM dithiothreitol, 5 mM magnesium chloride, 80 mM potassium chloride, 2% glycerol, 0.25 nM unlabelled single-stranded oligonucleotide and 50 μg $ml^{-1}$ poly-(dI–dC). The single-stranded oligonucleotide was included to compete for the binding of unspecific proteins binding to single-stranded DNA. Two microlitres of loading buffer (25% Ficoll 400, 0.25% bromophenol blue) was added. Binding of STAT-5 was analysed on a 4% polyacrylamide gel in 0.25 × TBE. Prerun and electrophoresis of 3 h were each performed at room temperature at 10 V $cm^{-1}$ with recirculation of electrophoresis buffer.

### RESULTS

### Effects of retinoids on expression of PRL-R mRNA

We detected PRL-R mRNA expression in all four cell lines evaluated. Expression level was highest in T47D cells, lower in MCF-7 and SKBR-3 and lowest in BT-20 cells (results not shown). Major PRL-R mRNA transcripts of 13.7, 3.4 and 2.6 kb were detected in the T47D cell line (Figure 1A). Treatment of the breast cancer cell lines with 1 μM of four different retinoic acid analogues (ATRA, 9-*cis*-RA, CD2325 and CD437) for 24 h resulted in a significant reduction of PRL-R mRNA ($P<0.01$) (Figure 1A and B). The activity of the four different retinoids was statistically not distinguishable.

### Regulation of PRL-R mRNA in MCF-7 cells

Time- and dose-dependent regulation of PRL-R mRNA was observed (Figure 2A and B). A marked drop of message was detected even after treatment for only 1 h using 1 μM 9-*cis*-RA or after treating cells with $10^{-10}$ M 9-*cis*-RA for 24 h. To examine whether the retinoid-induced decrease in PRL-R expression was dependent on continuing protein synthesis, cycloheximide was introduced in our experiments (Figure 2C). Cycloheximide alone caused a slight decrease in mRNA expression to 90% of vehicle-treated control. Treatment with 9-*cis*-RA for 3 h decreased PRL-R mRNA levels to a similar extent in the absence and presence of cycloheximide, which demonstrated that, over this time frame, inhibition of expression by 9-*cis*-RA does not require continuing protein synthesis. Retinoids may also potentially destabilize PRL-R mRNA. To examine this possibility, cells were treated with the transcription inhibitor actinomycin D in the presence and absence of 1 μM 9-*cis*-RA, and the rate of the resulting decline in PRL-R mRNA levels was measured over a 12-h period (Figure 2D). The rate of decline in PRL-R mRNA levels was almost identical in vehicle- and RA-treated cells, which indicated that the decline in PRL-R mRNA levels could not be accounted for by a RA-induced destabilization of PRL-R mRNA.

### Regulation of PRL-R protein by 9-*cis*-RA in MCF-7 cells

To evaluate whether retinoid-induced suppression of PRL-R mRNA results in a diminished concentration of PRL-R protein on the cell surface, we performed radioligand binding assays. Treating cells for either 24 or 48 h with 1 μM 9-*cis*-RA resulted in at least a 40% decrease of PRL-R protein (Figure 3).

### Effects of pretreatment of T47D cells with 9-*cis*-RA on PRL-induced STAT-5 activation

To demonstrate that suppression of PRL-R mRNA and protein is also functionally important for PRL-dependent intracellular events, we studied modulation of STAT-5 activation, which is known to be triggered by the hormone-bound PRL-R. Using band-shift assays, a 15-min PRL pulse activates STAT-5. Pretreatment of T47D cells with 9-*cis*-RA for 1, 5 and 10 h suppresses PRL-mediated STAT-5 activation in a time-dependent fashion (Figure 4).

### DISCUSSION

This study showed that retinoids suppress PRL-R expression. PRL plays a major role in the induction and progression of mammary tumours in rodents (Welsch and Nagasawa, 1997; Wennbo et al, 1997) and in primates (Ng et al, 1997). In addition, human breast cancer cell lines regularly express PRL-R, and proliferation is induced by PRL in bovine lactogen-depleted culture medium (Biswas and Vonderhaar, 1987). In addition to its role as a lactogenic hormone, PRL is also known to trigger, together with other steroidal hormones, the proliferation of normal breast tissue, and has been described as activating the transcription of growth-related genes (Doppler et al, 1994). Most recently, it was reported that breast cancer cell lines synthesize and secrete biologically active PRL (Clevenger et al, 1995; Ginsburg and Vonderhaar, 1995). Therefore, an auto- or paracrine loop mediated by PRL and PRL-R may be involved in the regulation of proliferation of human breast cancer cells (Bhatavdekar and Patel, 1997; Shaw-Bruha et al, 1997). In organ culture experiments, this auto/paracrine effect of PRL could be confirmed (Wennbo et al, 1997). Looking for substances interfering with this autostimulating system, we found retinoids to down-regulate PRL-R mRNA and protein in breast cancer cell lines. The same effect has been observed for sodium butyrate (Ormandy et al, 1992) and phorbol ester (Ormandy et al, 1993), both substances known to be antiproliferative in breast cancer cell lines. RA and analogues are known to inhibit growth of breast cancer cells alone or

 © Cancer Research Campaign 1999

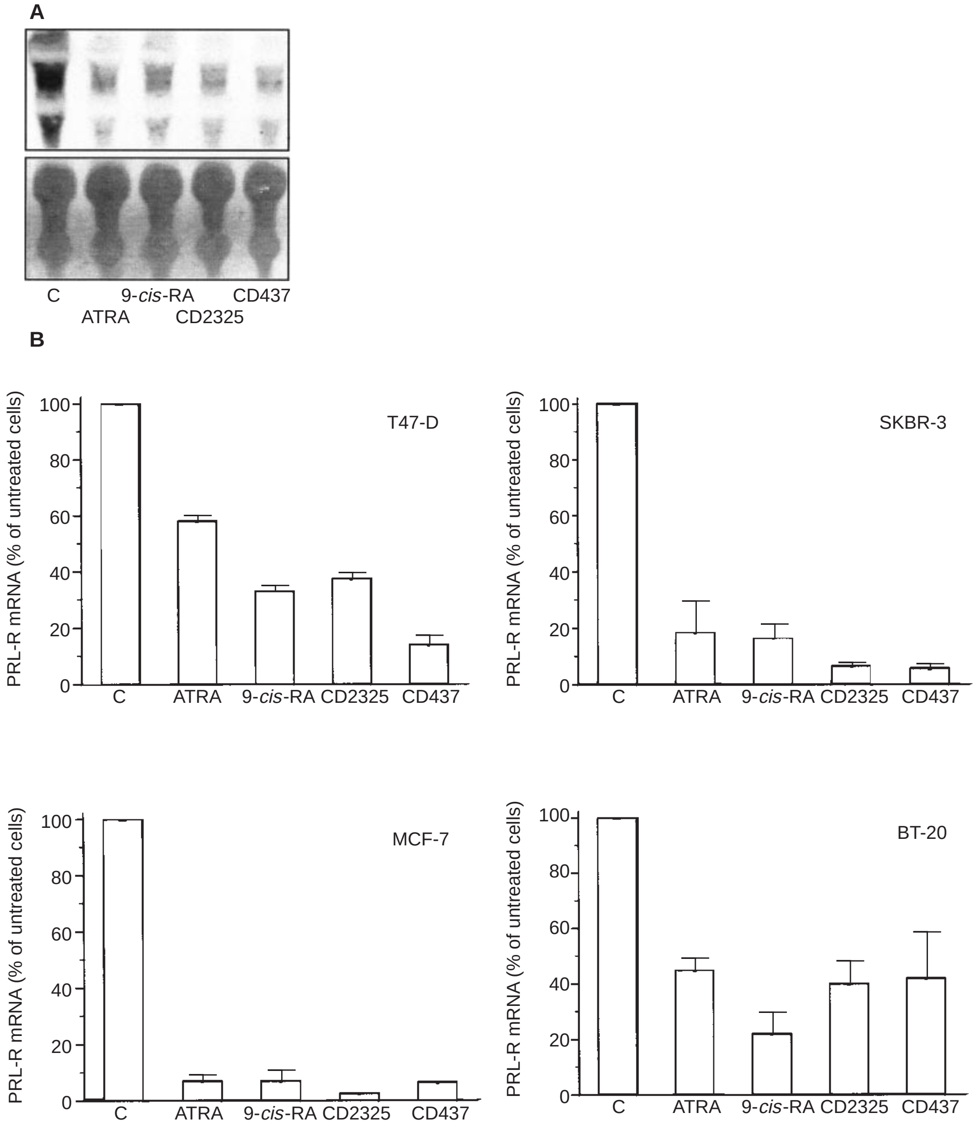

**Figure 1** Effects of retinoids on the expression of PRL-R mRNA in human breast cancer cell lines. (**A**) T47D cells were treated for 24 h either with the vehicle as control or with the retinoids indicated (1 μM). Northern blotting was performed as described in the Materials and methods section. Staining of total RNA with ethidium bromide (shown at the bottom of the blot) confirmed the integrity of RNA and showed comparable RNA loading in each lane. The data are representative of three seperate experiments. (**B**) Cells were treated for 24 h either with the vehicle as control or with the retinoids indicated (1 μM). Reverse transcription polymerase chain reaction (RT-PCR) and quantification were performed as described in Materials and methods. Data are expressed as percentages of vehicle-treated controls. Each bar represents the mean value of three independent experiments; error bars represent the s.e.m. of triplicate determinations in combination with other biological response modifiers amplifying their antiproliferative potency (Marth et al, 1986). Recently, we and others (Widschwendter et al, 1995; Fanjul et al, 1996) demonstrated RAR-γ to be involved in retinoid-mediated antiproliferative effects. Synthetic retinoid analogues CD437 and CD2325, which demonstrated RAR-γ selectivity and a strong antiproliferative potency in breast cancer cells (Shao et al, 1995; Widschwendter et al, 1997), showed the same effect on PRL-R expression as did natural substances.

In a paracrine autocrine loop, it is difficult to verify that the retinoid-mediated down-regulation of the PRL-R is responsible for the proliferation inhibition, but comparing PRL-R expression levels and responsiveness to RA treatment we could find the following: in this study, we used two oestrogen receptor-positive (T47D and MCF-7) and two oestrogen receptor-negative breast cancer cell lines (SKBR-3 and BT20). The SKBR-3 cell line is the only known oestrogen receptor-negative breast cancer cell line which is RA sensitive. The SKBR-3 and MCF-7 cell lines have similar PRL-R expression levels and response patterns to treatment with RA, whereas the second oestrogen receptor-negative cell line, BT-20, expresses the PRL-R at a very low level and is not responsive to treatment with RA. This fact supports our hypothesis that RA-mediated PRL-R down-regulation is in part responsible for the retinoid-mediated proliferation inhibition.

RA treatment, not only in vitro but also in vivo, was able to interfere with the tumour-stimulating activity of PRL: rats given

*© Cancer Research Campaign 1999* 

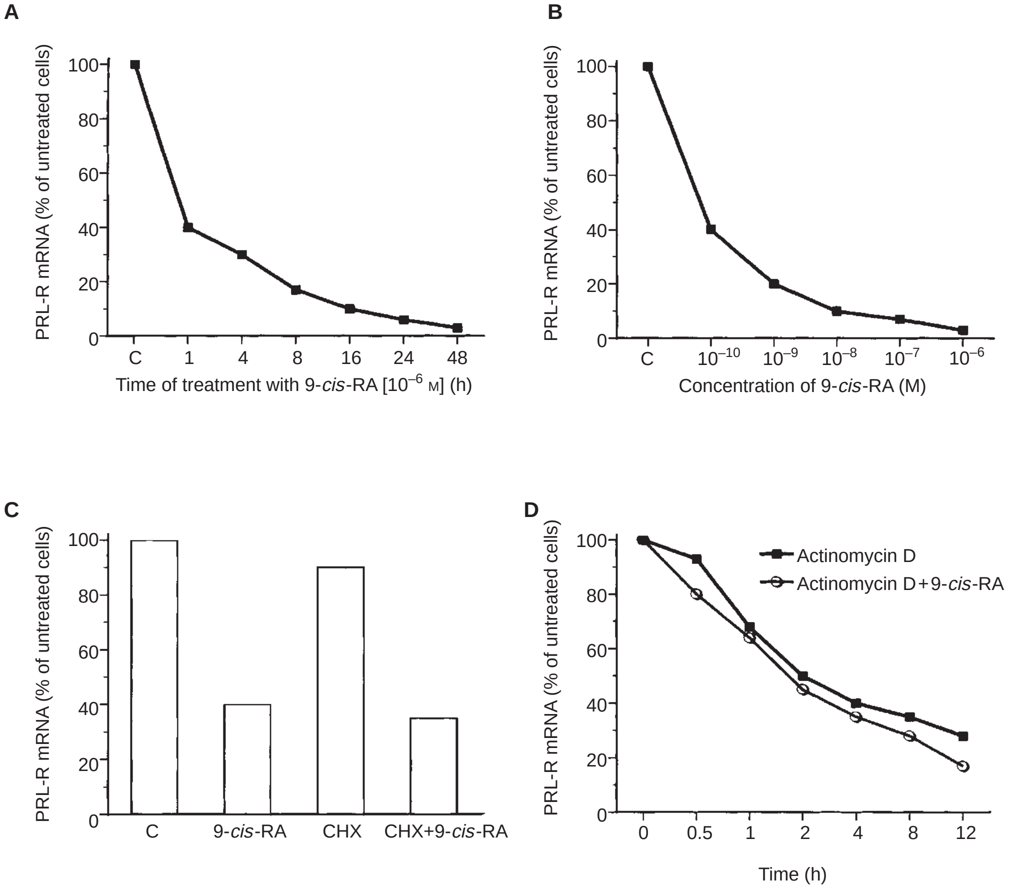

**Figure 2** Regulation of PRL-R mRNA expression in MCF-7 breast cancer cell line. (**A**) MCF-7 cells were treated with 1 μM 9-*cis*-RA for indicated times. (**B**) Dose dependency of the 9-*cis*-RA action after 24 h. (**C**) MCF-7 cells were treated for 3 h with vehicle, 1 μM 9-*cis*-RA, 20 μg $ml^{-1}$, CHX or with the combination 9-*cis*-RA and CHX. (**D**) MCF-7 cells were treated with 5 μg $ml^{-1}$ actinomycin D (ActD) and vehicle (■) or 5 μg $ml^{-1}$ ActD and 1 μM 9-*cis*-RA (○) for various times. PRL-R mRNA was measured as described in Materials and methods

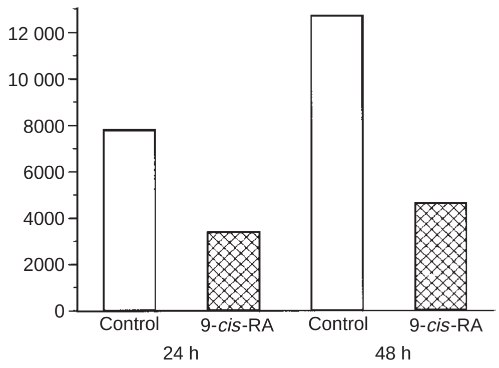

**Figure 3** Effects of 9-*cis*-RA on PRL-R protein expression in MCF-7 breast cancer cell line. MCF-7 cells were treated for 24 or 48 h with vehicle for control or 1 μM 9-*cis*-RA before measurement of PRL-R protein as described in the Materials and methods section. Results are expressed as c.p.m. $mg^{-1}$ protein

*N*-methyl-*N*-nitrosourea and subsequently treated with the prolactin secretion-stimulating drug haloperidol responded with a significant increase in mammary carcinoma development when compared with control rats. RA treatment of haloperidol-treated rats significantly ($P$<0.001) blocked the PRL-mediated stimulatory effect on mammary carcinoma development (Welsch et al, 1984). In this model, retinoid treatment has no effect on PRL serum levels, indicating that the anti-tumour effect of retinoids must occur at the level of tumour cells stimulated by PRL. Very recently, a paper was published by Ng et al (1997) in which they report the following: treatment of ageing monkeys with growth hormone (GH) resulted in a fourfold increase in mammary glandular size and epithelial proliferation index. GH activates the GH receptor and the PRL-R. The GH receptor was not detected in mammary epithelium, whereas the PRL-R concentrates in the mammary epithelium. This group could not distinguish whether GH could stimulate proliferation directly by acting through the epithelial PRL-R, or indirectly by increasing insulin-like growth factor I (IGF-I) which then acts through its cognate receptor in mammary epithelium. The theory that PRL-R mediates proliferation was very recently supported by Wennbo et al (1997). They showed – using transgenic mice overexpressing the bovine GH and mice overexpressing the rat PRL – that the PRL-R alone is sufficient for induction of mammary carcinomas in mice, whereas activation of the GH receptor is not sufficient for mammary

 © Cancer Research Campaign 1999

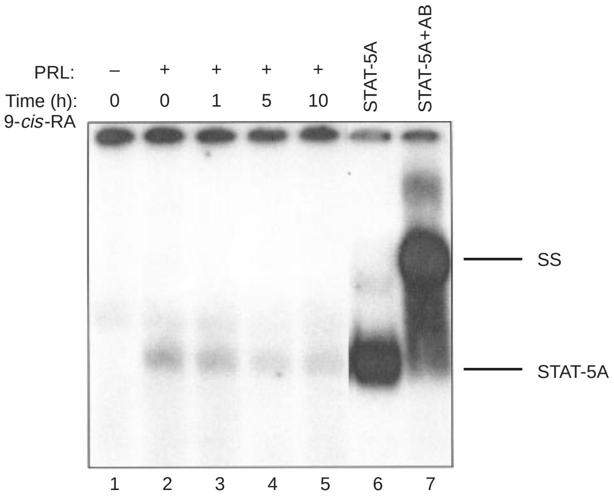

**Figure 4** Effects of 9-*cis*-RA pretreatment on STAT-5 induction. T47D cells were kept on confluency for 1 day. 9-*cis*-RA was added to the culture medium 1 h (lane 3), 5 h (lane 4) or 10 h (lane 5) before PRL stimulation (5 μg $ml^{-1}$, 15 min, lanes 2–5). Extracts were derived and equal amounts were compared in electrophoretic mobility shift assays. Lane 6, STAT-5A expressed in COS-7 cells (T. Welte and W. Doppler, unpublished data). Lane 7, supershift (ss) STAT-5A and anti-STAT-5A (antibody against the C-terminal end, Santa Cruz Biotechnology, CA, USA). The data are representative of two separate experiments tumour formation. These recent data gave, at least in part, some evidence that suppression of PRL-R by retinoids could play an important role in the prevention or treatment of breast cancer. The only strategy to test whether retinoic acid has an impact on the in vivo situation would be to treat the monkeys concomitantly with retinoic acid to measure breast size, proliferation index and PRL-R expression.

Suppression of PRL-R mRNA by retinoids will probably appear to be on the level of transcription because it occurs independent of protein de novo synthesis or modulation of mRNA stability. Sodium butyrate and phorbol ester also inhibit PRL-R gene expression by a transcriptional mechanism that does not require continuing protein synthesis (Ormandy et al, 1992, 1993). The mechanism of this transcriptional regulation is still unclear. A recent study by Møldrup et al (1996) showed that the PRL-R promoter contains a perfect repeat of a motif, AGGTCA, common among the nuclear receptors; it is separated by one nucleotide. Nuclear factors (e.g. hepatocyte nuclear factor 4) are thought to bind as homodimers to this element, often in competition with other nuclear receptors (RARs and RXRs). One attractive model explaining the rapid suppression of PRL-R, even at very low doses of RA, could be the interference between the above-mentioned nuclear pathways. To investigate the functional importance of this process, we studied the modulation of a PRL-dependent intracellular signal transduction pathway by retinoids, namely the activation of STAT-5, a factor known to be activated by PRL (Welte et al, 1994). Pretreatment of T47D cells with 9-*cis*-RA suppresses PRL-mediated STAT-5 activation, demonstrating a functional effect of RA-mediated down-regulation of PRL-R.

We have demonstrated that retinoids are able to down-regulate PRL-R in breast cancer cells. The functional importance of this process was shown by suppressing PRL-induced activation of STAT-5 by RA pretreatment. In view of previous data demonstrating PRL to be an important growth factor in breast cancer cells, we hypothesize that down-regulation of PRL-R is, in part, responsible for RA-dependent proliferation inhibition in breast cancer cell lines.

### ACKNOWLEDGEMENTS

We express our thanks to Dr Peter Berger and Dr Stephan Dirnhofer for helpful discussions, to Inge Gaugg and Martina Fleischer for excellent technical assistance, and to Dr Eugen Preuss for layout. We acknowledge Professor Bollag for providing 9-*cis*-RA.

This study was generously supported by a grant from the Austrian National Bank, project no. 5788 and by a grant from the Fonds zur Förderung wissenschaftlicher Forschung no. P12617-MED.

### REFERENCES

Anderson E, Ferguson JE, Morten H, Shalet SM, Robinson E and Howell A (1993) Serum immunoreactive and bioactive lactogenic hormones in advanced breast cancer patients treated with bromocriptine and octreotide. *Eur J Cancer* **29**: 209–217

Anzano MA, Byers SW, Smith JM, Peer CW, Mullen LT, Brown CC, Roberts AB and Sporn MB (1994) Prevention of breast cancer in the rat with 9-*cis* retinoic acid as a single agent and in combination with tamoxifen. *Cancer Res* **54**: 4614–4617

Bhatavdekar JN and Patel DD (1997) Ectopic prolactin: as a local growth promoter in breast cancer (abstract). In *Proceedings of 33rd Annual Meeting of the American Society of Clinical Oncology*, 1990, Vol. 16, *ASCO*

Biswas R and Vonderhaar BK (1987) Role of serum in prolactin responsiveness of MCF-7 human breast cancer cells in long term tissue culture. *Cancer Res* **47**: 3509–3514

Bonneterre J, Peyrat JP, Beuscart R, Lefebvre J and Demaille A (1987) Prognostic significance of prolactin receptors in human breast cancer. *Cancer Res* **47**: 4724–4728

Boutin JM, Edery M, Shirota M, Jolicoeur C, Lesueur L, Ali S, Gould D, Djiane J and Kelly P (1989) Identification of a cDNA encoding a long form of prolactin receptor in human hepatoma and breast cancer cells. *Mol Endocrinol* **3**: 1455–1461

Clevenger CV, Chang W-P, Ngo W, Pasha TLM, Montone KT and Tomaszewski JE (1995) Expression of prolactin and prolactin receptor in human breast carcinoma. *Am J Pathol* **146**: 695–705

Cobleigh MA, Dowlashahi K, Deutsch TA, Mehta RG, Moon RC, Minn F, Benson AB, Rademaker AW, Ashenhurst JB, Wade JL and Wolter J (1993) Phase I/II trial of tamoxifen with or without fenretinide, an analog of vitamin A, in women with metastatic breast cancer. *J Clin Oncol* **11**: 474–477

Costa A (1993) Breast cancer chemoprevention. *Eur J Cancer* **29A**: 589–592

Costa A, Formelli F, Chiesa F, Decensi A, De Palo G and Veronesi U (1994) Prospects of chemoprevention of human cancer with the synthetic retinoid fenretinide. *Cancer Res* **54**(suppl.): 2032S–2037S

Doppler W (1994) Regulation of gene expression by prolactin. *Rev Physiol Biochem Pharmacol* **124**: 92–130

Doppler W, Villunger A, Jennewein P, Brduscha K, Groner B and Ball RK (1991) Lactogenic hormone and cell type specific control of the whey acidic protein gene promoter in transfected mouse cells. *Mol Endocrinol* **5**: 1624–1632

Fanjul AN, Bouterfa H, Dawson M and Pfahl M (1996) Potential role for retinoic acid receptor-γ in the inhibition of breast cancer cells by selective retinoids and interferons. *Cancer Res* **56**: 1571–1577

Formelli F, Clerici M, Campa T, Gaetana Di Mauro M, Magni A, Mascotti G, Moglia D, De Palo G, Costa A and Veronesi U (1993) Five year administration of fenretinide: pharmacokinetics and effects on plasma retinol concentrations. *J Clin Oncol* **11**: 2036–2042

Ginsburg E and Vonderhaar BK (1995) Prolactin synthesis and secretion by human breast cancer cells. *Cancer Res* **55**: 2591–2595

Goffin V and Kelly PA (1996) Prolactin and growth hormone receptors. *Clin Endocrinol* **45**: 247–255

Gottardis MM, Lamph WW, Shalinsky DR, Wellstein A and Heyman RA (1996) The efficacy of 9-*cis* retinoic acid in experimental model of cancer. *Breast Cancer Res Treatment* **38**: 85–96

Gouilleux F, Wakao H, Mundt M and Groner B (1994) Prolactin induces phosphorylation of tyr694 of Stat5(MGF), a prerequisite for DNA binding and induction of transcription. *EMBO J* **13**: 4361–4368

© Cancer Research Campaign 1999

Koga M and Sutherland RL (1991) Retinoic acid acts synergistically with 1,25-dihydroxyvitamin D3 or antiestrogen to inhibit human breast cancer cell proliferation. *J Steroid Biochem Mol Biol* **39**: 455–460

Malarkey WB, Kennedy M, Allred LE and Milo G (1983) Physiological concentrations of prolactin can promote the growth of human breast tumor cells in culture. *J Clin Endocrinol Metab* **56**: 673–677

Marth C, Daxenbichler G and Dapunt O (1986) Synergistic antiproliferative effect of human recombinant interferons and retinoic acid in cultured breast cancer cells. *J Natl Cancer Inst* **77**: 1197–1202

Marth C, Widschwendter M and Daxenbichler G (1993) Mechanism of synergistic action of all-*trans*- or 9-*cis*-retinoic acid and interferons in breast cancer cells. *J Steroid Biochem Mol Biol* **47**: 123–126

Møldrup A, Ormandy C, Nagano M, Murthy K, Banville D, Tronche F and Kelly PA (1996) Differential promoter usage in prolactin receptor gene expression: hepatocyte nuclear factor 4 binds to and activates the promoter preferentially active in the liver. *Mol Endocrinol* **10**: 661–671

Moon RC and Mehta RG (1990) Chemoprevention of mammary cancer by retinoids. *Basic Life Sci* **52**: 213–224

Ng ST, Zhou J, Adesanya OO, Wang J, LeRoith D and Bondy CA (1997) Growth hormone treatment induces mammary gland hyperplasia in aging primates. *Nature Med* **3**: 1141–1144

Ormandy CJ, de Fazio A, Kelly PA and Sutherland RL (1992) Transcriptional regulation of prolactin receptor gene expression by sodium butyrate in MCF-7 human breast cancer cells. *Endocrinology* **131**: 982–984

Ormandy CJ, Lee CSL, Kelly PA and Sutherland R (1993) Regulation of prolactin receptor expression by the tumour promoting phorbol ester 12-*o*-tetradecanoylphorbol-13-acetate in human breast cancer cells. *J Cell Biochem* **52**: 47–56

Rui H, Kirken RA and Farrar WL (1994) Activation of receptor-associated tyrosine kinase JAK2 by prolactin. *J Biol Chem* **269**: 5364–5368

Sachs L (1992) *Angewandte Statistik*, pp. 380–392. Springer-Verlag: Berlin

Shao Z-M, Dawson MI, Su Li X, Rishi AK, Sheikh MS, Han Q-X, Ordonez JV, Shroot B and Fontana JA (1995) p53 independent $G_0/G_1$ arrest and apoptosis induced by a novel retinoid in human breast cancer cells. *Oncogene* **11**: 493–504

Shaw-Bruha CM, Pirrucello SJ and Shull JD (1997) Expression of the prolactin gene in normal and neoplastic human breast tissues and human mammary cell lines: promoter usage and alternative mRNA splicing. *Breast Cancer Res Treatment* **44**: 243–253

Sporn MB, Roberts AB and Goodman DS (1994) *The Retinoids*: *Biology, Chemistry and Medicine*, 2nd edn. Raven Press: New York

Tso JH, Sun X-H, Kao T, Reece KS and Wu R (1985) Isolation and characterization of rat and human glyceraldehyde-3-phosphate dehydrogenase c-DNA: genomic complexity and molecular evolution of the gene. *Nucl Acids Res* **13**: 2485–2502

Vonderhaar BK (1989) Estrogens are not required for prolactin induced growth of MCF-7 human breast cancer cells. *Cancer Lett* **47**: 105–110

Welsch CW and Nagasawa H (1997) Prolactin and murine tumorigenesis: a review. *Cancer Res* **37**: 951–963

Welsch CW, DeHoog JV, Scieszka KM and Aylsworth CF (1984) Retinoid feeding, hormone inhibition, and/or immune stimulation and the progression of *N*-methyl-*N*-nitrosourea-induced rat mammary carcinoma: suppression by retinoids of peptide hormone-induced tumor cell proliferation in vivo and in vitro. *Cancer Res* **44**: 166–171

Welte T, Garimorth K, Philipp S and Doppler W (1994) Prolactin-dependent activation of a tyrosine phosphorylated DNA binding factor in mouse mammary epithelial cells. *Mol Endocrinol* **8**: 1091–1102

Wennbo H, Gebre-Medhin M, Gritli-Linde A, Ohlsson C, Isaksson OGP and Törnell J (1997) Activation of the prolactin receptor but not the growth hormone receptor is important for induction of mammary tumors in transgenic mice. *J Clin Invest* **100**: 2744–2751

Widschwendter M, Daxenbichler G, Dapunt O and Marth C (1995) Effects of retinoic acid and interferon-γ on expression of retinoic acid receptor and cellular retinoic acid binding protein in breast cancer cells. *Cancer Res* **55**: 2135–2139

Widschwendter M, Daxenbichler G, Bachmair F, Müller E, Zeimet AG, Windbichler G, Uhl-Steidl M, Lang T and Marth C (1996) Interaction of retinoic acid and interferon-α in breast cancer cell lines. *Anticancer Res* **16**: 369–374

Widschwendter M, Daxenbichler G, Culig Z, Michel S, Zeimet AG, Mörtl MG, Widschwendter A and Marth C (1997) Activity of retinoic acid receptor-γ selectively binding retinoids alone and in combination with interferon-γ in breast cancer cell lines. *Int J Cancer* **71**: 497–504

*© Cancer Research Campaign 1999*
